# Failure Evaluation of Bridge Deck Based on Parallel Connection Bayesian Network: Analytical Model

**DOI:** 10.3390/ma14061411

**Published:** 2021-03-15

**Authors:** Yang Ding, Jingliang Dong, Tonglin Yang, Shuangxi Zhou, Yongqi Wei

**Affiliations:** 1 Department of Civil Engineering, Zhejiang University, Hangzhou 310058, China; 2 Quanzhou Institute of Equipment Manufacturing Haixi Institutes, Chinese Academy of Sciences, Quanzhou 362000, China; 3 School of Civil Engineering and Architecture, East China Jiao Tong University, Nanchang 330013, China; green.55@163.com; 4 College of Chemistry and Chemical Engineering, Hunan University, Changsha 410082, China; 5 School of Materials Science and Engineering, Tongji University, Shanghai 201804, China; wei_yongqi@tongji.edu.cn

**Keywords:** bridge deck, failure, Monte Carlo, Bayesian network

## Abstract

Failure is a major element that causes deterioration, which in turn affects the serviceability of long span bridges. Currently, the Bayesian network, which relates to probability statistics, is widely used for evaluating fatigue failure reliability. In particular, Bayesian network can not only calculate the fatigue failure at the system level, but also deduce the fatigue failure at the weld level. In this study, a system-level fatigue reliability evaluation model of a bridge deck (BD), which is seen as a parallel system, is proposed based on the Bayesian network. A fatigue probability reliability model of the BD was derived using the master S-N curve. In addition, the Monte Carlo (MC) method was applied to solve the multi-dimensional and complex analytical expressions in the Bayesian network. The applicability of the proposed model was demonstrated by three numerical case studies.

## 1. Introduction

Fatigue cracks are generated at the welds of a bridge deck (BD) in long span bridges, that is, welds at the rib to deck and rib to diaphragm, under combined traffic load [1,2], welding residual stress [3], and environmental effects [4], which can be destructive [5]. Therefore, it is necessary to evaluate the performance of the BD in long span bridges to ensure reliable fatigue-resistant design [6,7,8,9,10,11].

In particular, fatigue behavior may generally be characterized by the so-called S-N curve, which allows the fatigue life under a given stress level to be predicted [12,13,14,15,16,17]. For example, Cui et al. (2020) presented a framework for fatigue damage prognosis of the BD in long span cable stayed bridges based on the S-N curve [18]. Jiang et al. (2019) established the crack initiation fatigue life and loss of strength fatigue life model of rectangular hollow section welds with and without concrete infill and perfobond ribs based on the S-N curve [19]. Clearly the S-N curve method can accurately predict the fatigue life based on the above research results [20]. However, deterministic models fail to account for uncertainty inherent in the monitoring data and interpretation of the model error [21]. Therefore, probabilistic models based on S-N curve are required.

Bayesian methods are widely used to describe uncertainty [22,23]. Chiachio et al. (2014) presented a Bayesian prediction approach as a general method to incorporate modeling uncertainties for inference about the damage process [24]. The uncertainty of the welds’ parameters can be quantified by the Bayesian model. Furthermore, the Bayesian network is an extension of the Bayesian model used to address interactions between the welds of the system, such as the BD, which includes rib to deck welds and rib to diaphragm welds [25,26]. Gehl et al. (2016) estimated the probability of occurrence of failure modes (i.e., various configurations of weld damage states) using a Bayesian network at the system level [27].

The main contributions of the current work are: (1) a system-level fatigue reliability evaluation model of the BD is proposed based on the Bayesian network; and (2) a fatigue probability reliability model of the BD is derived using the master S-N curve. Three numerical case studies were adopted to verify the applicability of the proposed model. In the numerical case studies, the influence of the probability distribution of the stress amplitude (SA) and cycle number (CN) on fatigue life reliability is analyzed. Compared with the deterministic method, the uncertainty of random parameters is considered; in addition, the fatigue performance of the BD can be evaluated. The remainder of this paper is structured as follows. The probabilistic fatigue reliability equation is derived based on the master S-N curve, and development of the Bayesian network for the BD at weld and system levels are described in Section 2. The results of weld- and system-level evaluation of fatigue reliability of the BD based on three numerical case studies are given in Section 3. Finally, Section 4 presents the conclusion, which summarizes the results of this paper.

## 2. Development of Bayesian Network for the BD at System Level

### 2.1. Probabilistic Model of Fatigue Reliability

The deterministic fatigue model is widely used to evaluate the reliability of long span bridges, high rise structures, wind turbines, etc., and is based on the master S-N curve [28,29]. In the deterministic fatigue model, the parameters are considered as constants, thereby neglecting the potential for uncertainty due to instrument error and environment influence [30]. To evaluate the fatigue reliability more accurately, the fatigue probability analytical expressions of the master S-N curve were derived as expressed in Equation (1), which considers the parameters as variable (refer to Appendix A):

(1) $$S_{e}=CN^{h}$$

The Palmgren Miner linear cumulative rule is widely used to evaluate fatigue damage during a period of time, and can be expressed by [31]

(2) $$D=\sum_{i=1}^{n}\frac{n_{i}}{N_{i}}=\frac{n_{1}}{N_{1}}+\frac{n_{2}}{N_{2}}+\cdots +\frac{n_{n}}{N_{n}}\;n_{i}=n_{tot}f\left(S_{e}\right)S_{e}\;n_{tot}=\sum_{i=1}^{n}n_{i}$$

Furthermore, the expression of the fatigue damage *D* can be rewritten based on the definition of the limit, that is, the sum of the cumulative superposition can be calculated in integral form, which can be expressed by (refer to Appendix A):

(3) $$D=\underset{\Delta S_{e}\to 0}{\lim}\sum_{i=1}^{n}\frac{n_{i}}{N_{i}}=\underset{\Delta S_{e}\to 0}{\lim}\sum_{i=1}^{n}\frac{n_{tot}f\left(S_{e}\right)S_{e}}{N_{i}}\to \int_{S_{e}}\frac{n_{tot}f(S_{e})}{N}dS_{e}$$

Then, the service life can be calculated based on the fatigue damage balance equation, which can be expressed by [32]:

(4) $$g(D_{f},S_{e},C,n_{tot},t)=D_{f}-t\times D=0$$

Finally, the fatigue failure probability *p_f_*, and the fatigue reliability index *β* can be calculated based on the probability theory, which can be expressed by (refer to Appendix A):

(5) $$p_{f}=P\left\{g(D_{f},S_{e},C,n_{tot},t)\leq 0\right\}=P\left\{D_{f}-t\times \int_{0}^{\infty }\frac{n_{tot}f(S_{e})C^{1/h}}{S_{e}^{1/h}}dS_{e}\leq 0\right\}\;\beta =\Phi^{-1}(1-p_{f})=-\Phi^{-1}(p_{f})$$

### 2.2. Construction of the Bayesian Network for the BD

Generally, Bayes’ theorem can describe the correlation between two events, as first proposed by Thomas Bayes, and can be seen as the following equation [33]:

(6) $$P(A|S)=\frac{P(A)P(S|A)}{P(S)}$$

In particular, the Bayesian network is proposed to deal with multi-event problems when there are a large number of events, that is, S = {A_1_, A_2_, … A*_i_*}, which is an extension of the Bayesian model (refer to Appendix A). That is, the interactions between the welds of the system and the correlation between the welds and the system can be calculated based on the Bayesian network, which can be expressed by:

(7) $$p(S)=p\left\{A_{1},\cdots ,A_{n}\right\}=\prod_{i=1}^{n}p(A_{i}\left|M_{i}\right.)\;p(A_{i})=\sum_{{\mathrm{except}\;A}_{i}}p(S)$$

Generally, the BD is always seen as a parallel system, that is, the system is defined to fail when all of the nodes fail, which can be expressed as [34]:

*P*(S = 1 | A = 1, B = 0) = 0 *P*(S = 1 | A = 0, B = 1) = 0 *P*(S = 1 | A = 1, B = 1) = 1 (8)

where the number 1 is represents failure and the number 0 represents no failure.

The failure probability of A, B, and S and the failure sequence of A and B can be inferred based on the Bayesian theorem, which can be expressed by [35]:

(9) $$P((B=1|A=1)|S=1)=\frac{P(B=1|A=1,S=1)}{P(S=1)}\;P((A=1|B=1)|S=1)=\frac{P(A=1|B=1,S=1)}{P(S=1)}\;P((A=1,B=1)|S=1)=\frac{P(A=1,B=1,S=1)}{P(S=1)}\;\begin{array}{l} P(S=1)=P(S=1|(B=1|A=1))\times P(B=1|A=1)\\ +P(S=1|(A=1|B=1))\times P(A=1|B=1)\\ +P(S=1|(A=1,B=1))\times P(A=1,B=1) \end{array}$$

where B = 1|A = 1 describes B failure after A failure; A = 1|B = 1 describes A failure after B failure; A = 1, B = 1 describes simultaneous failure of A and B.

Furthermore, the failure probability of the system S can be calculated based on the probability theory. Then, Equation (9) can be rewritten as:

(10) $$\begin{array}{l} P_{f}(S=1)=P_{f}(D_{A}\leq D_{fA},D_{B}\leq D_{fB})+P_{f}(D_{A}\leq D_{fA},D_{fB}\leq D_{B}\leq D_{fS})\\ +P_{f}(D_{B}\leq D_{fB},D_{fA}\leq D_{A}\leq D_{fS}) \end{array}$$

### 2.3. Computation Algorithm of the Proposed Bayesian Network

To solve the Bayesian network, it must be solved in the form of integra [36]:

(11) $$E\left[g(x)\right]=\int g(x)p(x)dx$$

The Monte Carlo (MC) method has increasingly been adopted to solve the complicated, intractable, and multi-dimensional integration in the Bayesian network, and uses a large number of samples to approximate the expectation of a target distribution [37]. That is, a set of samples *x*(*t*) are obtained from the target distribution *p*(*x*) and then the expectations of the target distribution can be calculated based on the summation of these samples, which can be expressed by [38]:

(12) $$E\left[g(x)\right]=\frac{1}{n}\sum_{t=1}^{n}g(x^{\left(t\right)})\underset{n\to \infty }{\to }\int g(x)p(x)dx$$

As shown in Equation (12), the expectation can be approached with summation over a large number of samples, that is, the accuracy of the solution depends on the number of samples *n*, based on the strong law of large numbers without analytic integration (refer to Appendix A).

## 3. Numerical Case Study

**Figure 1:**
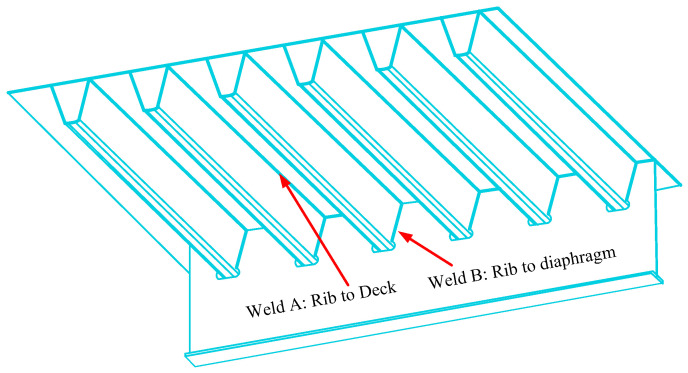
Bridge deck.

As can be seen from Figure 1, fatigue prone welds include rib to deck welds, which can be represented by A, and rib to diaphragm welds, which can be represented by B. Furthermore, there are three numerical case studies, that is, different distributions of the SA and CN, for two welds, as seen in Table 1. For the numerical case study 1, the SA and CN of A are the same as for B. For the numerical case study 2, the SA and CN of A are higher than for B. For the numerical case study 3, the SA of A is higher than for B, but the CN of A is lower than for B.

**Figure 2:**
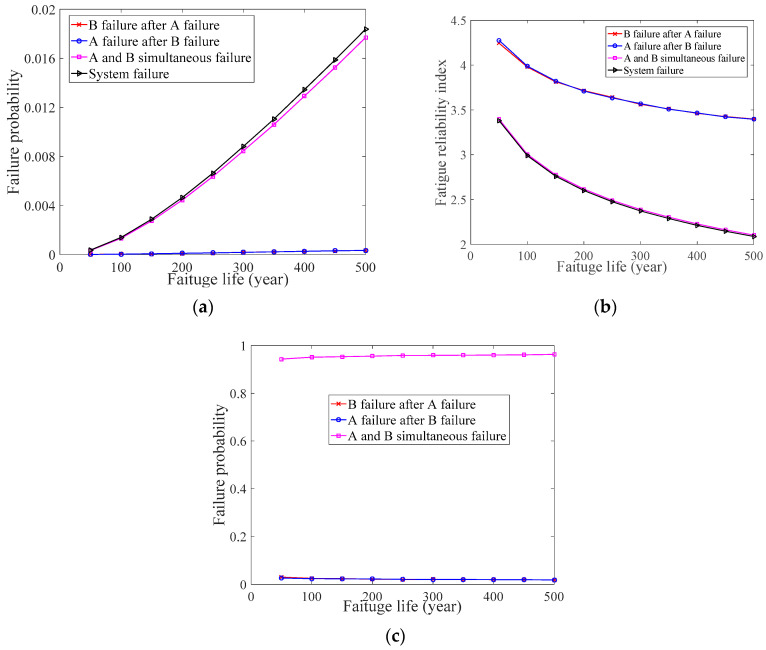
Numerical case study 1. (**a**) Fatigue probability of the welds and system; (**b**) Fatigue reliability index of the welds and system; (**c**) Bayesian inference.

In the numerical case study 1, the value of A and B simultaneous failure’s *p_f_* is the same as the system’s *p_f_*, whereas the values of B failure after A failure’s *p_f_* and A failure after B failure’s *p_f_* are almost zero, which can be seen in Figure 2a. Similarly, the value of B failure after A failure’s *β*, A failure after B failure’s *β*, A and B simultaneous failure’s *β*, and the system’s *β* also shows this change, which can be seen in Figure 2b. Furthermore, when the value of the system’s *p_f_* is known, the value of B failure after A failure’s *p_f_*, A failure after B failure’s *p_f_*, and A and B simultaneous failure’s *p_f_* can be inferred based on Bayes’ theorem. As can be seen in Figure 2c, the value of A and B simultaneous failure’s *p_f_* is almost one, that is, the system’s *p_f_* can be described by the A and B simultaneous failure’s *p_f_*. Finally, we can determine that A and B are important welds on the BD, that is, the fatigue performance of A and B needs to be considered simultaneously to describe the change in the BD’s *p_f_*.

**Figure 3:**
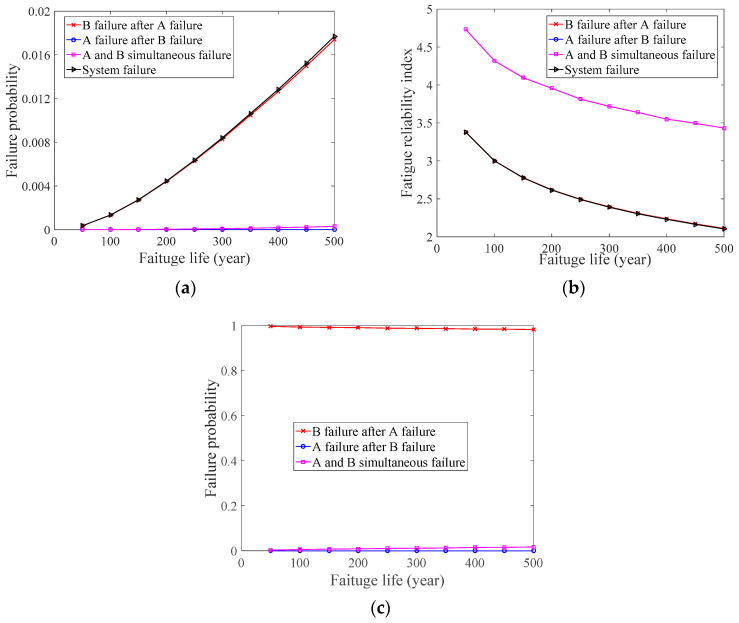
Numerical case study 2. (**a**) Fatigue probability of the welds and system; (**b**) Fatigue reliability index of the welds and system; (**c**) Bayesian inference.

In the numerical case study 2, the value of B failure after A failure’s *p_f_* is the same as the system’s *p_f_*, whereas the values of A and B simultaneous failure’s *p_f_* and A failure after B failure’s *p_f_* are almost zero, which can be seen in Figure 3a. Similarly, the value of B failure after A failure’s *β*, A failure after B failure’s *β*, A and B simultaneous failure’s *β*, and the system’s *β* also shows this change, which can be seen in Figure 3b. Furthermore, when the value of the system’s *p_f_* is known, the value of B failure after A failure’s *p_f_*, A failure after B failure’s *p_f_*, and A and B simultaneous failure’s *p_f_* can be inferred based on Bayes’ theorem. As can be seen in Figure 3c, the value of B failure after A failure’s *p_f_* is almost one, that is, the system’s *p_f_* can be described by B failure after A failure’s *p_f_*. Finally, we can determine that A is an important weld on the BD, that is, the fatigue performance of A needs to be considered to describe the change in the BD’s *p_f_*.

**Figure 4:**
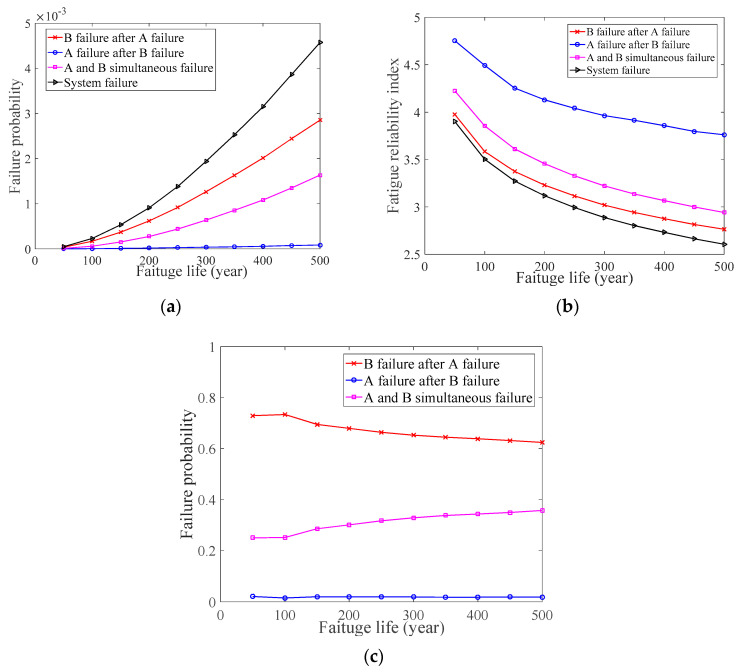
Numerical case study 3. (**a**) Fatigue probability of the welds and system; (**b**) Fatigue reliability index of the welds and system; (**c**) Bayesian inference.

In the numerical case study 3, the value of B failure after A failure’s *p_f_*, A failure after B failure’s *p_f_*, A and B simultaneous failure’s *p_f_*, and the system’s *p_f_* are different from each other, which can be seen in Figure 4a. Similarly, the value of the fatigue reliability index of B failure after A failure, A failure after B failure, A and B simultaneous failure, and system failure also shows this change, which can be seen in Figure 4b. Furthermore, when the value of the system’s *p_f_* is known, the value of B failure after A failure’s *p_f_*, A failure after B failure’s *p_f_*, and A and B simultaneous failure’s *p_f_* can be inferred based on Bayes’ theorem. As can be seen in Figure 4c, B failure after A failure’s *p_f_* and A and B simultaneous failure’s *p_f_* have a significant influence for the system’s *p_f_*. Finally, we can determine that A is an important weld on the BD and that B will fail quickly after A failure, that is, the fatigue performance of A and B needs to be considered simultaneously to describe the change in the BD’s *p_f_*.

## 4. Conclusions

This paper presents a system fatigue reliability evaluation model of the BD based on the Bayesian network. Based on the master S-N curve, the fatigue probability reliability model of the BD, which includes rib to deck welds and rib to diaphragm welds, was derived. For the Bayesian network, the random variables, which include the SA and NC, are correlated. Furthermore, when the failure probability of the system is known, the failure sequence of the welds, that is, rib to deck welds and rib to diaphragm welds, can also be calculated based on the Bayesian network. In addition, the Monte Carlo method was applied to solve the multi-dimensional and complex analytical expressions in the proposed model. The main conclusions of this paper are as follows: (1) By comparison of the results of three numerical case studies, the fatigue reliability of the BD was the highest when the distribution of the two welds’ stress amplitudes and cycle numbers were the same. Therefore, the traffic flow should be controlled as much as possible to ensure that the stress amplitude and cycle number of the two welds on the BD are similar. In future research, the fatigue reliability of the BD system with multiple welds will be analyzed and the method will be verified by experiments. (2) Based on the Bayesian network method, the fatigue reliability of the system can be derived from the fatigue reliability of welds. Similarly, the fatigue reliability of welds can be deduced from the known fatigue reliability of the system.

## Figures and Tables

**Table 1:** Probability distribution of random variables in three numerical case studies.

Numerical Case Study	Welds	*S_d_*		*n_d_*	
		*μ*	*σ*	*μ*	*σ*
Numerical case study 1	A	ln (10)	0.1	ln (3000)	0.05
	B	ln (10)	0.1	ln (3000)	0.05
Numerical case study 2	A	ln (10)	0.1	ln (3000)	0.05
	B	ln (5)	0.1	ln (2000)	0.05
Numerical case study 3	A	ln (10)	0.1	ln (1000)	0.05
	B	ln (5)	0.1	ln (5000)	0.05

## Data Availability

Data is contained within the article.

## Appendix A

*C* is constant for a given material, *C* = 19,930.2;
$S_{e}$ is the stress amplitude (SA);
*N* is the total cycle number (CN);
*n_i_* is the CN for the *n*th cycle;
*f*(*S_e_*) is the expression of the SA;
*n_tot_* is the total CN.
*t* is service life;
*D_f_* follows the lognormal distribution *LN* (0, 0.294);
*p_f_* is the fatigue failure probability;
*β* is fatigue reliability index;
*D_f_**_i_* is the failure damage of the weld *i*;
*x* is an unknown parameter;
*g*(*x*) is the function of *x*;
*p*(*x*) is the target function of *x*;
*E*[] is the mathematical expectation.
